# Role of TLR4 as a prognostic factor for survival in various cancers: a meta-analysis

**DOI:** 10.18632/oncotarget.24178

**Published:** 2018-01-12

**Authors:** Bo Hao, Zhen Chen, Baochen Bi, Miaomei Yu, Shuang Yao, Yuehua Feng, Yang Yu, Lili Pan, Dongmei Di, Guanghua Luo, Xiaoying Zhang

**Affiliations:** ^1^ Department of Cardiothoracic Surgery, The Third Affiliated Hospital of Soochow University, Changzhou 213003, P.R. China; ^2^ Department of Urology, The Third Affiliated Hospital of Soochow University, Changzhou 213003, P.R. China; ^3^ Comprehensive Laboratory, The Third Affiliated Hospital of Soochow University, Changzhou 213003, P.R. China

**Keywords:** Toll like receptor 4, solid tumor, prognosis, meta-analysis

## Abstract

**Background:**

Accumulating evidence showed that high expression of toll like receptor 4 (TLR4) was significantly associated with the outcome of patients with solid cancers. However, other studies failed to draw a similar conclusion. Thus, a systematic meta-analysis was performed to assess the prognostic value of TLR4 in solid tumors.

**Results:**

Data from 15 studies and 1294 patients were enrolled. Among the 15 studies, 14 studies demonstrated the association between overall survival(OS) and TLR4 expression, and 7 studies described the relationship between disease-free survival(DFS) and TLR4 expression. High expression of TLR4 was significantly associated with poor OS (pooled hazard ratio (HR) = 2.05; 95% confidence interval (CI) (1.49, 2,49), *P* < 0.001). The results of meta regression analysis indicated that the subgroups of ethnic (PD = 0.924), tumor type (PD = 0.669), HR obtained method (PD = 0.945), analysis type (PD = 0.898), and cut-off value(PD = 0.835) were not the resource of heterogeneity. Moreover, patients with elevated TLR4 had a significantly worse DFS (pooled HR = 1.79; 95% CI (1.11, 2.88), *P* < 0.05).

**Materials and Methods:**

We searched PubMed, Embase and the Cochrane Library (last update by April 18, 2017) to identify literatures evaluating the value of TLR4 in cancer patients. Combined hazard ratios (HRs) for OS and DFS were assessed using fixed-effects models and random effects models respectively.

**Conclusions:**

The meta-analysis suggests that elevated expression of TLR4 is associated with poor OS and shorter DFS of patients with solid tumors. The results indicate that TLR4, as a novel prognostic biomarker in solid tumors, could potentially help to improve treatment decision-making of solid tumors in clinical.

## INTRODUCTION

The increasing morbidity and mortality of cancer and its impact on social public health have gained much attention, and numerous researches exploring the mechanisms of occurrence, development and metastasis of cancers have been conducted [1]. Although targeted therapies and comprehensive treatments for some cancers have made rapid progress, the outcome of the vast majority of cancer patients still remain poor. Thus, a useful biomarker which is able to predict the prognosis of cancers is urgently needed.

Chronic inflammation is reported to be closely associated with tumors [2, 3], and recent studies have found that, various mechanisms, under the condition of chronic inflammation, could facilitate development and progression of carcinoma, including activating angiogenesis, inhibiting apoptosis, stimulating cell proliferation and survival, inducing gene mutations, subverting antitumor immune responses [4, 5], and inducing epigenetic alterations closely related to cancer development. A large amount of cell signaling pathways have been investigated and discovered, and an increasing number of evidence has shown that toll like receptors contribute significantly to solid malignancy.

Currently, newly discovered evidences constantly proved that innate immune system plays an important role during the procedure of angiogenesis in cancer tissues. It can synthesize angiogenic factors which will cause endothelial cell recruitment, proliferation and new vessel formation [6, 7]. All the pathological process mentioned above will eventually lead to tumor promotion. [6–10]. TLRs are a family of transmembrane receptors that are best-known for their role in host defence against infection. TLRs prevent the entry of pathogens by activating pathogen-associated molecular patterns (PAMPs), which are produced by microorganisms, as well as endogenous macromolecules produced by damaged tissues [11]. It is clearly established that TLR4 stimulates PAMPs to defend against invading exogenous pathogens and recognizes the endogenous ligands from necrotic cells through damage-associated molecular patterns (DAMPs) [12]. Activating the TLR4 expressed on tumor cells to promote tumor cell survival, and upregulate the expression of nuclear factor-kappa B (NF-κB) and antiapoptotic proteins [13]. Sato et al. [14] deemed that the expression of the DAMP-derived molecules was upregulated in the tumor microenvironment and caused TLR4-related chronic inflammation, leading to carcinogenesis, cancer progression, and metastasis. TLR4 signaling is reported to be related to numerous cancers, such as lung [15], liver [16], gastric [17], pancreatic [18], ovarian [19], and colon cancer [20], all the cancers above are generally believed to have some sort of link with local chronic inflammation.

Emerging evidence have demonstrated that increased expression of TLR4 is closely related to poor OS and worse DFS in solid cancer patients [21–24]. However, Wei et al. found that high serum TLR4 was associated with the better outcome of early-stage NSCLC patients [25]. Some researchers have shown that high TLR4 expression is related to the prognosis of the malignant diseases, while others failed to come to a similar conclusion. Therefore, a meta-analysis was conducted to assess the prognostic value of upregulated TLR4 in cancer patients.

## RESULTS

### Study characteristics

**Figure 1:**
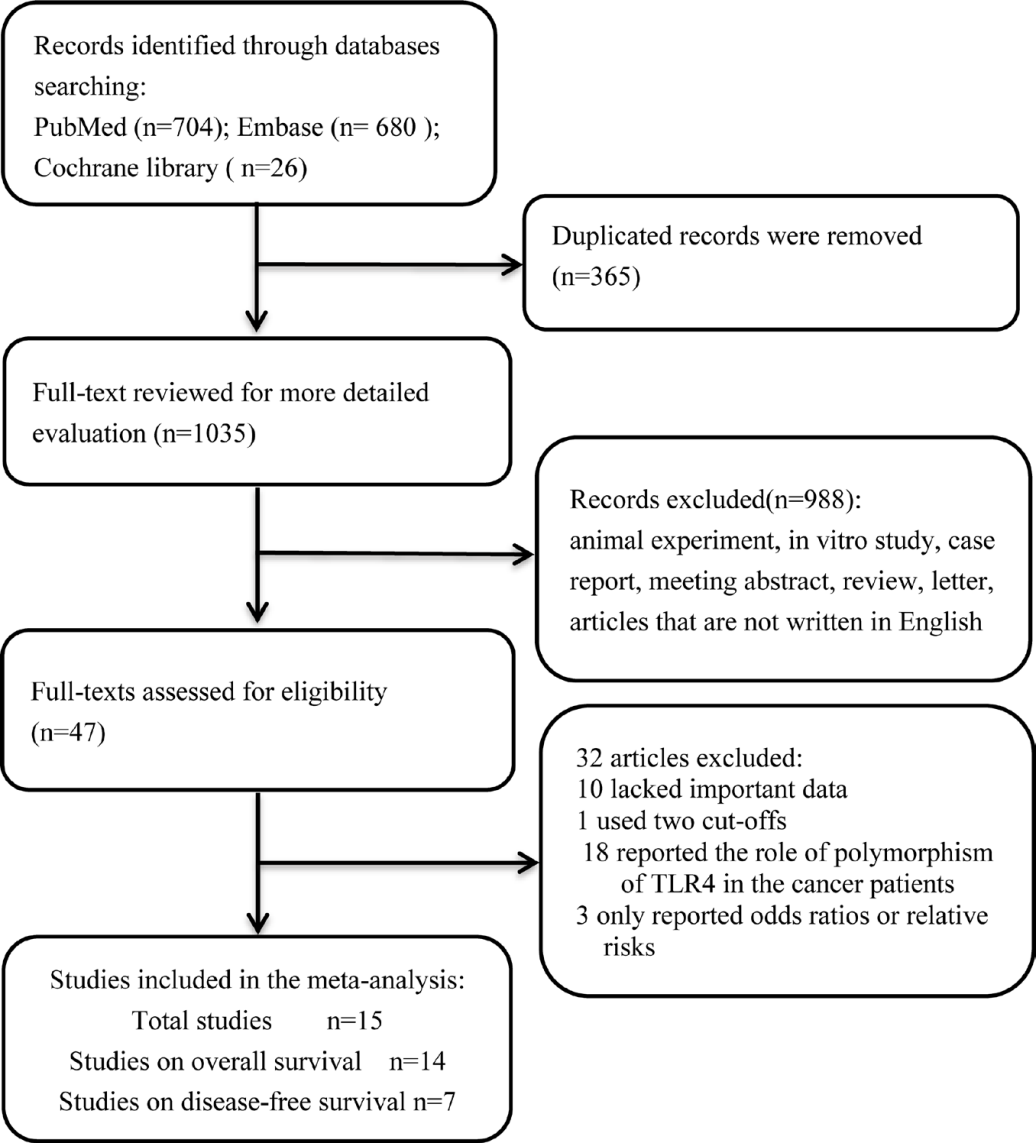
Flow diagram of the study selection process

By the initial search (Figure 1), a total of 1035 literatures were retrieved. After scanning the titles, abstracts, types and full text of the above publications, then 988 literatures were excluded (lack of relation, review, letter, comment, studies on cancer cell lines and experimental animal researches and articles that were not written in English). After carefully reading the articles, 32 were excluded (10 lacked some important data, 1 used two cut-offs, 18 investigated the role of polymorphism of TLR4 in prognosis of various cancers and 3 only reported odds ratios or relative risks). Finally, a total of 15 studies [16, 18, 21–33] were included in our meta-analysis. The main characteristics of all enrolled studies were shown in the Table 1. Among the studies, 14 studies demonstrated the associations between overall survival and TLR4 expression, and 7 studies described the relations between disease-free survival and TLR4 expression. A total of 1347 patients from Italy, Spain, China, Ireland, Japan and South Korea were diagnosed with various cancers, including non-small cell lung cancer, epithelial ovarian cancer, colorectal cancer, oral squamous cell carcinoma, hepatocellular carcinoma, breast cancer and pancreatic ductal adenocarcinoma. Among the 14 studies for OS, 11 studies reported on Asian, and 3 studies reported on Caucasian. Among the 7 studies for DFS, 6 studies reported on Asian and 1 study reported on Caucasian. There were 4 studies providing HRs directly in the text, and other 11 studies providing the survival curve. The cut-off values were different in each study.

**Table 1:** Main characteristics of all studies included in the meta-analysis

Study	Country	Tumor type	Case number	Gender (M/F)	TNM stage	Detection method	Follow-up (months)	Survival analysis	Cut-off value	Multivariate analysis	HR
Cammarota 2010	Italy	CRC	53	21/32	NR	IHC	108	DFS	> 20% of cells stained	no	SC
Chen 2015	China	BC	60	0/60	41/19(I-II/III)	IHC	Median 21	OS	IRS≥6	no	SC
Eiro 2013	Spain	CRC	104	60/44	7/63/18/16 (Duke A/B/C/D)	IHC	Mean148	OS	≥ 10% of cells stained	no	SC
d’Adhemar 2014	Ireland	EOC	85	0/85	18/5/46/6(FIGO I/II/III/IV)	IHC	NR	OS	IRS>4	no	SC
Jing 2012	China	HCC	106	88/18	16/60/30 (T1/T2/T3)(UICC)	IHC	Over 60	OS	> 30% of cells stained	no	SC
Eiró2013	Spain	HCC	30	25/5	NR	IHC	Over 60	OS	IRS>0	no	SC
Kim 2012	South Korea	EOC	123	0/123	54/8/44/17 (FIGO I/II/III/IV)	IHC	Mean 43	OS	IRS≥4	no	SC
Ma 2014	China	BC	205	0/205	NR	IHC	Median 98	OS/DFS	IRS≥4	yes	report
Ren 2013	China	OSCC	61	41/20	11/20/12/18 (I/II/III/IV)( AJCC)	IHC	Median 46	OS	> 30% of cells stained	no	SC
Wang 2010	Japan	CRC	108	62/46	I-II/III/IV(36/44/28)	IHC	60	OS/DFS	> 30% of cells stained	yes	report
Wang 2017	China	NSCLC	126	70/56	31/34/61 (I/II/III)	IHC	Median 36	OS/DFS	IRS ≥ 6	yes	report
Wei 2016	China	eNSCLC	28	17/11	28 (I-II)	ELISA	Over 60	OS/DFS	stage I 33.8ng/mL, stage II 48.9ng/mL	no	SC
Yang 2016	China	OSCC	110	89/21	37/73 (I-II/III-IV)	IHC	Over 60	OS/DFS	IRS ≥ 4	no	SC
Zhang 2010	China	PDAC	65	40/25	24/41(I-II/III-IV)	IHC	Median 14	OS	> 10% of cells stained	no	SC
Zhu 2012	China	EOC	83	0/83	24/41(I-II/III-IV)	IHC	Over 60	OS/DFS	> 30% of cells stained	no	report

Abbreviation: BC, breast cancer; HCC, hepatocellular carcinoma; EOC, epithelial ovarian cancer; CRC, colorectal cancer; OSCC, oral squamous cell carcinoma; NSCLC, non-small cell lung cancer; eNSCLC, early-stage non-small cell lung cancer; PDAC, pancreatic ductal adenocarcinoma; IHC, immunohistochemistry; ELISA, enzyme-linked immuno sorbent assay; HR, hazard ratio; IRS, immunoreactivity score; CI, confidence intervals; OS, overall survival; DFS, disease-free survival; HR hazard ratio; NR, not report; SC, survival curve; FIGO, International Federation of Gynecology and Obstetrics; AJCC, American Joint Committee on Cancer; UICC, Union for International Cancer Control; TNM, tumor lymph nodes metastasis; M, male; F, female.

### Quality assessment

Every eligible study enrolled in the meta-analysis was evaluated based on the Newcastle-Ottawa Quality Assessment Scale (NOS) [34]. The quality of all enrolled studies varied from 6 to 9, with a median of 7. Due to the score of all literatures greater than 6, all of them were included in our meta-analysis.

### Overall survival

**Figure 2:**
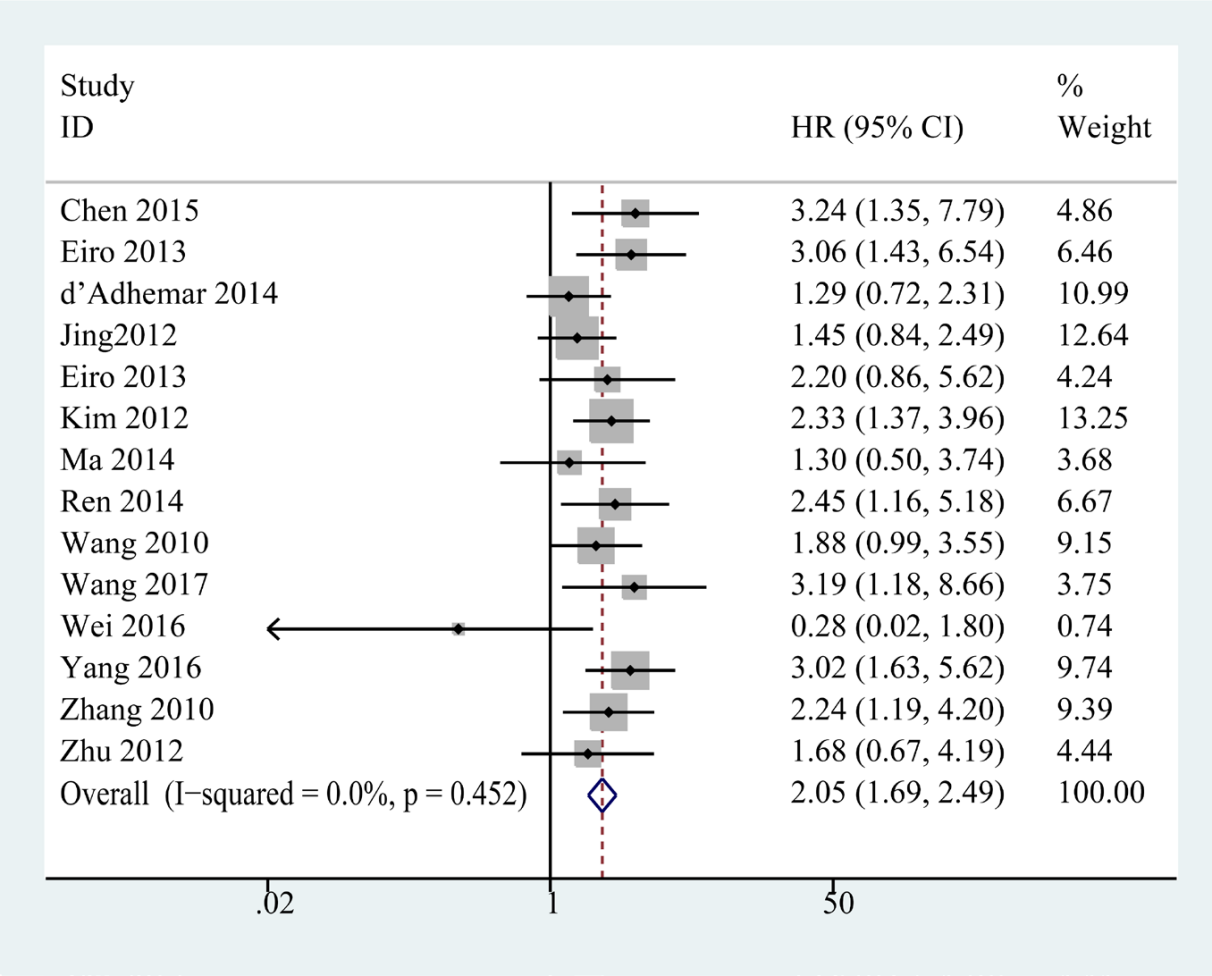
Forest plots of studies evaluating hazard ratios of high expression of TLR4 in solid tumors for overall survival

In our meta-analysis evaluating the effect of TLR4 expression on overall survival, there was no significant heterogeneity among those 14 studies (I^2^ = 0.0%, *P* = 0.451), and thus, a fixed-effects model was used to pool the HRs. As the results shown in Figure 2, an increased expression of TLR4 in cancer patients yielded a poor OS (pooled hazard ratio (HR) = 2.05, 95% CI (1.69, 2.49), *P* < 0.001).

**Figure 3:**
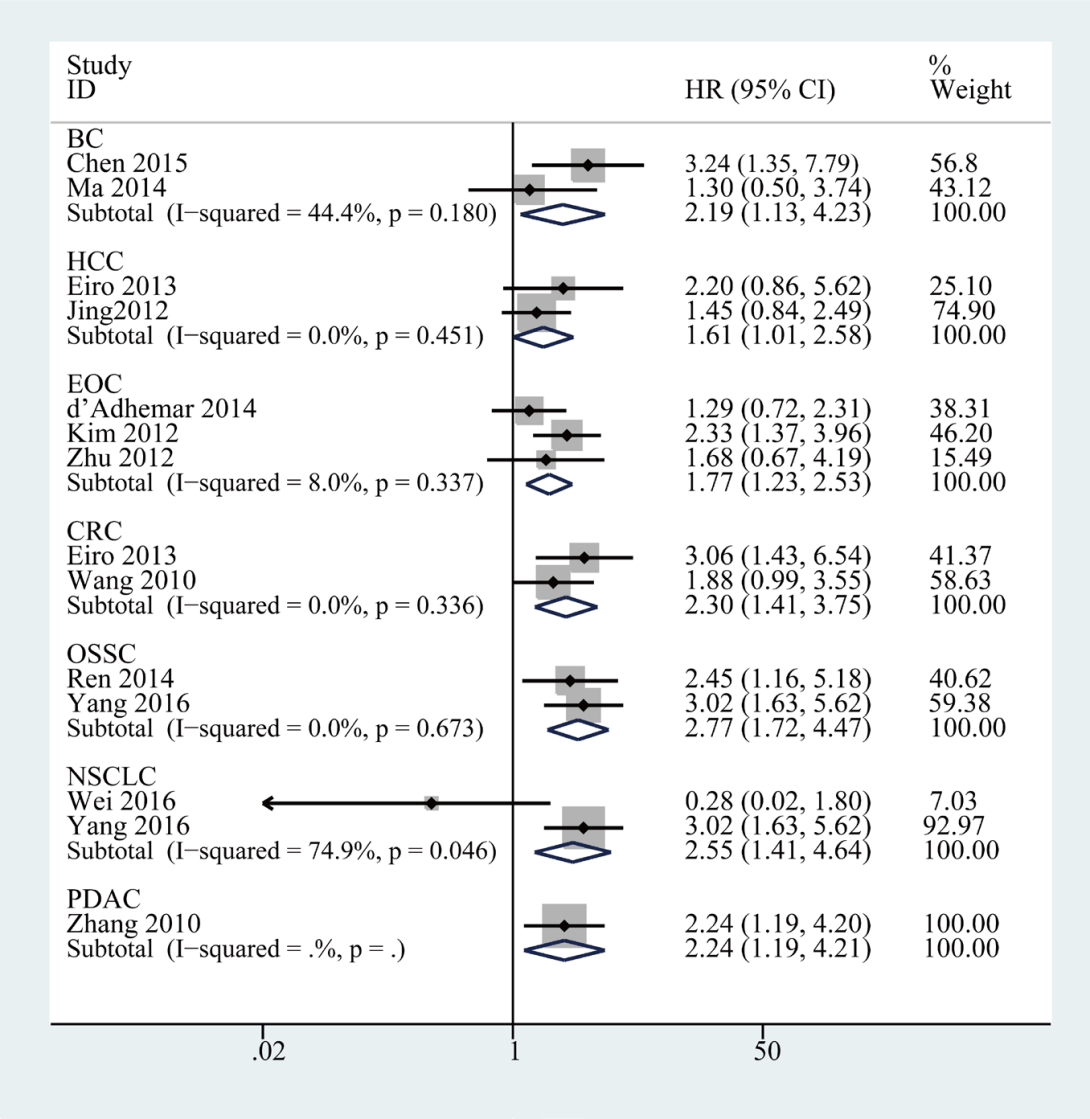
Forest plots of studies evaluating hazard ratios of elevated TLR4 expression for different tumor types

Further, the role of TLR4 in OS was investigated via subgroup analysis based on the main features, including ethnic lines, tumor type, HR obtain method, analysis type, and cut-off value. In the bgroup of ethnicity, increased TLR4 expression was an adverse predictor for OS in both Asian patients (HR = 2.11, 95% CI (1.69, 2.62), *P* < 0.001) and Caucasian patients (HR = 1.85, 95% CI (1.22, 2.80), *P* = 0.004) (Table 2). In the tumor type subgroup (Figure 3 and Table 2), we found the high expression of TLR4 was closely related to worse OS in breast cancer (combined HR = 2.19, 95% CI (1.13, 4.23), *P* < 0.05), hepatocellular carcinoma (combined HR = 1.61, 95% CI (1.01, 2.58), *P* < 0.05), epithelial ovarian cancer(combined HR = 1.77, 95% CI (1.23, 2.53), *P* < 0.05), oral squamous cell carcinoma(combined HR = 2.77, 95% CI (1.72, 4.47), *P* < 0.001) and colorectal cancer (combined HR = 2.30, 95% CI (1.41, 3.75), *P* < 0.01). There was only one study evaluating the relationship between TLR4 and OS in pancreatic ductal adenocarcinoma (HR = 3.85, 95% CI (2.27, 6.53); *P* < 0.05). However, the association between TLR4 and the prognosis of patients with non-small lung cancer was not significant in the analysis. The association between higher expression of TLR4 and poor OS outcome was statistically significant in other subgroups, including univariate analysis (HR = 2.07, 95% CI (1.67, 2.56), *P* < 0.001), multivariate (HR = 1.95, 95% CI (1.21, 4.14), *P* < 0.01), IRS ≥ 4 (HR = 2.36, 95% CI (1.63, 3,44), *P* < 0.001), > 30% of cells stained (HR = 1.77, 95% CI (1.26, 2.48), *P* < 0.01), others (HR = 2.10, 95% CI (1.55, 2.85), *P* < 0.001), reported in text (HR = 1.89, 95% CI (1.24, 2.88), *P* < 0.01) and Data-extrapolated (HR = 2.09, 95% CI (1.68, 2.60), *P* < 0.01).

**Table 2:** The pooled associations between different situations of TLR4 expression and the prognosis of patients with solid tumors

Outcome group	NO. of studies	No. of patients	HR (95% CI)	*P* value	PD	Model	Heterogeneity	
							I^2^	*P*
Overall survival	14	1294	2.05 (1.69, 2.49)	< 0.001		Fixed	0.0	0.452
Ethnicity					0.924			
Caucasian	3	219	1.85 (1.22, 2.80)	0.004		fixed	39.1%	0.194
Asian	11	1075	2.11 (1.69, 2.62)	< 0.001		fixed	0	0.497
Tumor type					0.669			
BC	2	265	2.19 (1.13, 4.23)	0.020		fixed	44.4%	0.180
HCC	2	136	1.61 (1.01, 2.58)	0.047		fixed	0	0.451
EOC	3	291	1.77 (1.23, 2.53)	0.002		fixed	8%	0.337
CRC	2	212	2.30 (1.41, 3.75)	0.001		fixed	0	0.336
OSCC	2	171	2.77 (1.72, 4.47)	< 0.001		fixed	0	0.673
NSCLC	2	154	1.19 (0.12,11.57)	0.882		random	74.9%	0.046
PDAC	1	65	3.85 (2.27, 6.53)	0.012		-	-	-
Analysis type					0.945			
Univariate	11	855	2.07 (1.67, 2.56)	< 0.001		fixed	11.7%	0.333
Multivariate	3	439	1.95 (1.21, 3.14)	0.006		fixed	0	0.455
Cut-off value					0.898			
IRS≥4	3	438	2.36 (1.63, 3.44)	< 0.001		fixed	0	0.375
>30% of cells stained	4	358	1.77 (1.26, 2.48)	0.001		fixed	0	0.732
Others	7	498	2.10 (1.55, 2.85)	< 0.001		fixed	28.4%	0.212
HR obtained method					0.835			
Report in text	4	378	1.89 (1.24, 2.88)	0.003		fixed	0	0.647
Data-extrapolated	10	916	2.09 (1.68, 2.60)	< 0.001		fixed	19.0%	0.268

Abbreviation: BC, breast cancer; HCC, hepatocellular carcinoma; EOC, epithelial ovarian cancer; CRC, colorectal cancer; OSCC, oral squamous cell carcinoma; NSCLC, non-small cell lung cancer; PDAC, pancreatic ductal adenocarcinoma; HR, hazard ratio; CI, confidence intervals; P_D_, P for subgroup difference.

**Figure 4:**
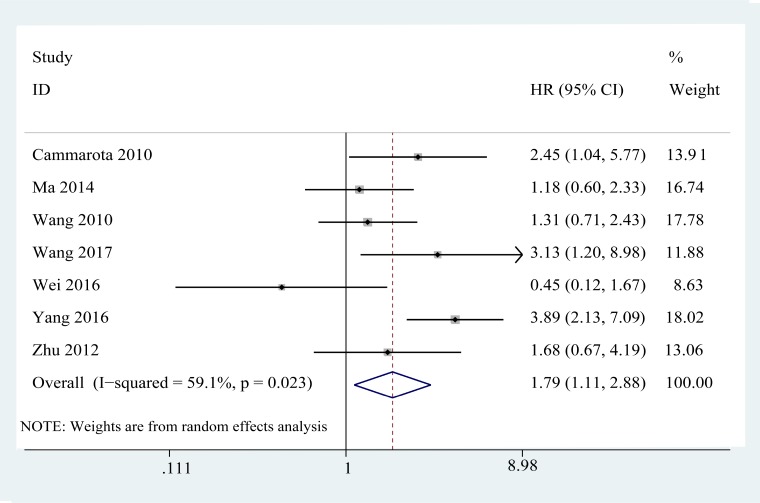
Forest plots of studies evaluating hazard ratios of increased expression of TLR4 in various cancers for disease-free survival

The results of meta regression analysis indicated that the subgroups of ethnic lines (PD = 0.924), tumor type(PD = 0.669), HR obtained method (PD = 0.945), analysis type(PD = 0.898), and cut-off value (PD = 0.835) were not the resource of heterogeneity.

**Figure 5:**
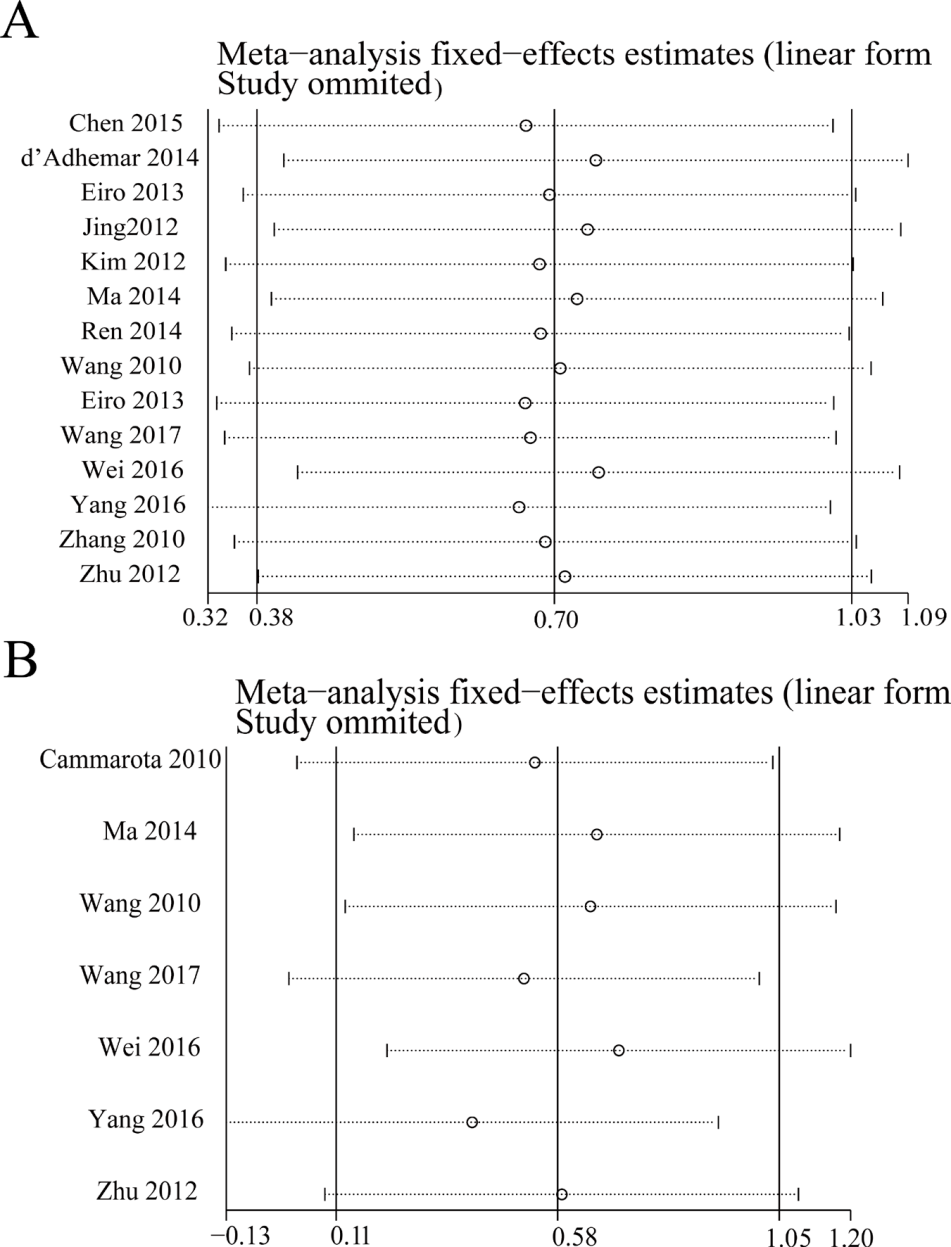
Sensitivity analysis for overall survival (**A**) and disease-free survival (**B**).

Each independent study for OS enrolled in our meta-analysis was deleted respectively to check if individual study influenced the results. Results of sensitivity analyses suggested that the findings were robust (Figure 5A).

**Figure 6:**
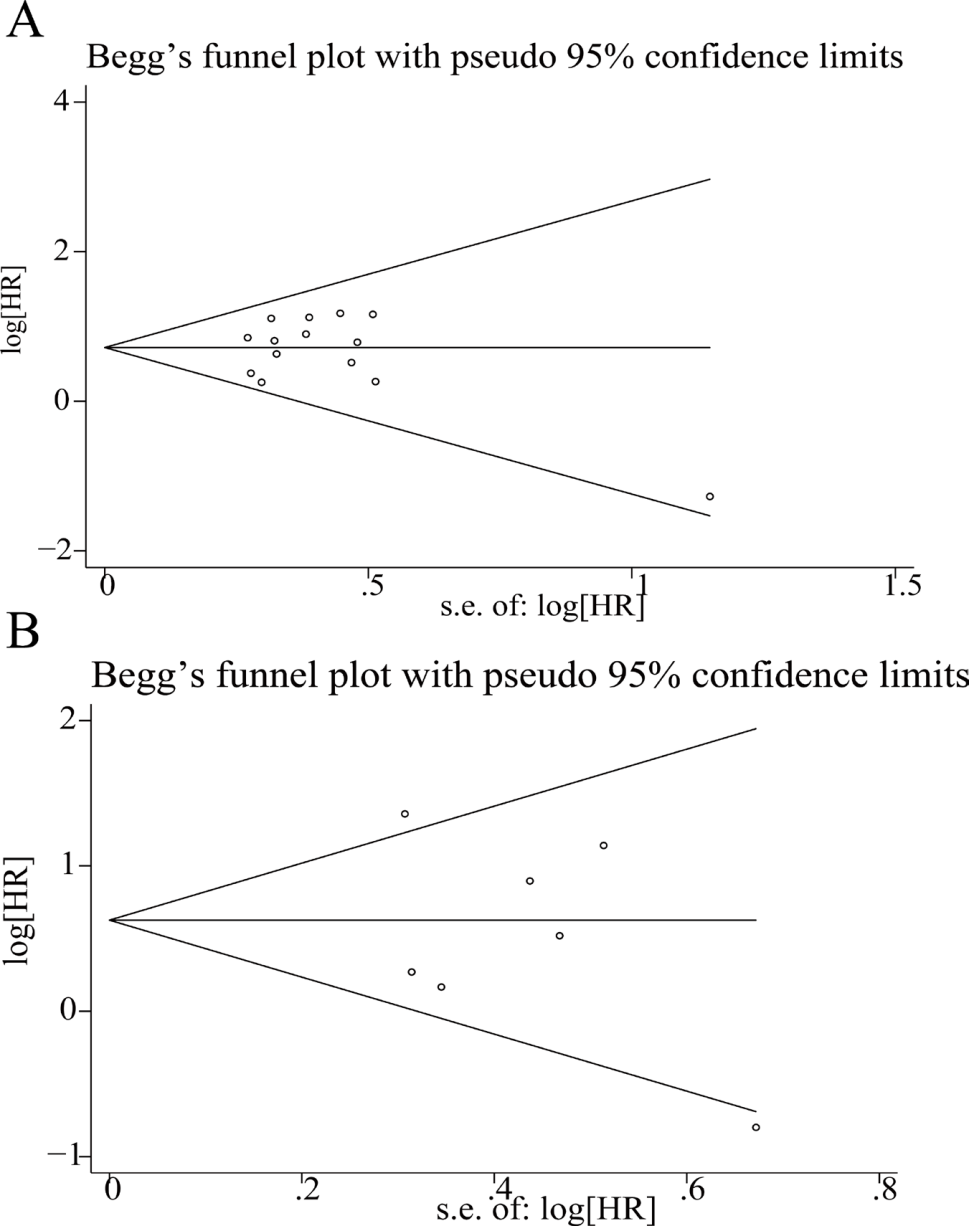
Funnel plots for the evaluation of potential publication bias (**A**) for overall survival and (**B**) for disease-free survival.

The publication bias of all included studies for OS was assessed with funnel plots, and Egger’s and Begg’s test. As shown in Figure 6A, the funnel plots were nearly symmetric. In Egger’s and Begg’s test, *P* > 0.05 (OS, *P* = 0.511 for the Begg’s test, *P* = 0.590 for the Egger’s test). Therefore, there did not exist significant publication bias among the included studies for OS.

### Disease-free survival

Seven studies [21–26, 33], enrolling a total of 713 patients, investigated the association between the DFS of 5 types of cancers and TLR4 expression level. Due to the presence of obvious heterogeneity (I^2^ = 59.1%; *P* = 0.023) among the studies (Figure 4), a random-effects model was applied. As the results shown in the Table 3, the pooled HR revealed a significant association between high level of TLR4 and poor DFS (pooled HR = 1.79, 95% CI (1.11, 2.88); *P* = 0.017).

**Table 3:** The pooled associations between TLR4 expression and disease-free survival

Outcome group	Studies	Patients	HR (95% CI)	*P* value	Model	Heterogeneity	
						I^2^	*P*
Disease-free survival	7	713	1.79 (1.11, 2.88)	0.017	random	59.1%	0.023
Ethnicity							
Asian	6	660	1.69 (0.97, 2.92)	0.064	random	64.9%	0.014
Caucasian	1	53	2.45 (1.04, 5.77)	0.040	-	-	-
Tumor type							
CRC	2	161	1.62 (0.98, 2.67)	0.058	fixed	26.1%	0.245
NSCLC	2	154	1.25 (0.19, 8.31)	0.297	random	81.0%	0.022
OSCC	1	110	3.89 (2.13, 7.10)	0.628	-	-	-
BC	1	205	1.18 (0.60, 2.32)	*P* < 0.001	-	-	-
EOC	1	83	1.68 (0.67, 4.20)	0.267	-	-	-
HR obtained method							
Reported in text	4	522	1.50 (1.03, 2.18)	0.037	fixed	0	0.429
Data-extrapolated	3	191	1.88 (0.65, 5.41)	0.244	random	76.6%	0.014

Abbreviation: BC, breast cancer; EOC, epithelial ovarian cancer; CRC, colorectal cancer; OSCC, oral squamous cell carcinoma; NSCLC, non-small cell lung cancer; HR, hazard ratio.

After stratification of the studies into subgroups based on Ethnicity, we found overexpression of TLR4 was significantly associated with a worse DFS in Caucasian populations (HR = 1.69, 95% CI (0.69, 2.07); *P* < 0.05), while the difference between elevated expression of TLR4 and DFS in Asian populations was not significant. However, there is only one study investigating the association between expression of TLR4 and DFS in Caucasian populations. Therefore, the conclusion needs to be confirmed in a larger sample of Caucasian population. In the subgroup of tumor type, the combined analysis indicated that the increased expression of TLR4 was significant associated with a poor DFS in breast cancer (HR = 1.18, 95% CI (0.60, 2.32); *P* < 0.001). However, in other cancers including colorectal cancer, non-small cell lung cancer, oral squamous cell carcinoma and epithelial ovarian cancer, the association was not significant. Restricting the analysis to studies that HRs were reported in the text, the results revealed a significant association between high expression of TLR4 and poor DFS (HR = 1.50, 95% CI (1.03, 2.18); *P* < 0.05), without heterogeneity (I^2^ = 0, *P* = 0.429). After combining the HRs obtained from survival curve, the analysis showed that there was not a significant association between the high TLR4 expression and a worse DFS.

The sensitivity analysis was conducted by sequential omission of individual studies using the fixed-effects model to check if individual study influenced the results. The result pattern was not obviously impacted by any single study (Figure 5B). The results of the funnel plot indicated that there was not any evidence of obvious publication bias among studies for DFS (*P* = 0.548 for the Begg’s test; *P* = 0.222 for the Egger’s test; Figure 6B).

## DISCUSSION

TLRs, as a family of pattern recognition receptors (PRRs), are capable of activating a variety of PAMPs and interacting with other families of PRRs which leads to a series of signal transduction [35]. Subsequently, various inflammatory mediators are produced, which are an important part of innate immunity, and ultimately, the acquired immune system is activated. The consequences of inflammatory immune response have two aspects: on the one hand, it makes organisms able to defend against infection; on the other hand, the continued inflammation environment could facilitate tumor cell immune escape [8]. Chronic inflammation promotes tumor cells to release various cytokines, leading to an inflammatory microenvironment and facilitating the occurrence and progression of tumors [2]. Moreover, tumor cells can also secrete cytokines, attracting inflammatory cells to infiltrate into tumors; as a result, infiltrated inflammatory cells secrete proteolytic enzymes and cytokines that can promote the proliferation of tumor cells, facilitating the formation of blood vessel, and enhancing capacity of metastasis of tumor cells [4, 36]. Up to now, at least 12 different TLRs which possess various ligands have been found and among which, TLR4 is the major receptor activated by lipopolysaccharide [37]. The signals mediated by TLR4 are transduced via two major pathways: one through the adapter protein myeloid differentiation factor 88 (MyD88), and the other through the TIR-domain-containing adapter-inducing interferon-β protein [38]. However, to date, TLR4 is the only ligand that is able to activate both MyD88 dependent and independent pathways. Upon activated by TLR4, MyD88 initiates the transcription of a specific set of genes involved in proinflammatory, antiviral and antibacterial responses [39, 40]. Both the MyD88 dependent and independent pathways promote NF-κB activation, leading to production of inflammatory cytokines [38]. Abnormal stimulation of the TLR4/NF-κB signaling pathway has been reported to be involved in numerous autoimmune diseases and chronic inflammation, and NF-κB have been reported to be highly expressed in several malignancies, and high expression of NF-κB was closely associated with the metastasis of carcinoma [24, 41]. Numerous studies indicated that NF-κB is a key regulator of Snail expression that plays a key role in cancers, especially in the metastasis of carcinoma [42–44]. Jing et al. [27] demonstrated that the activation of NF-κB up-regulated Snail expression in liver cancer cells, which facilitate those cells to undergo an EMT toward an invasive, metastatic tumor phenotype.

Recently, TLR4 was reported to be highly expressed in cancers, including colon cancer [20, 26], pancreatic ductal adenocarcinoma [18], oral squamous cell carcinoma [31], ovarian epithelial cancer [28], non-small lung cancer [33] and hepatocyte carcinoma [27]. And downregulation of TLR4 not only inhibits the tumor growth and cell colony formation in cancers [45–47], but also suppresses the metastasis of carcinoma [48]. Consistent with the above observations, activation of TLR4 promotes the invasion and metastasis of cancer cells [49, 50]. However, Ahmed et al. [51] observed that silencing of TLR4 promote tumor progression and metastasis in a murine model of breast cancer.

According to the statistical results, the combined risk of high TLR4 expression for OS in patients suffering from cancers was significant with a combined HR of 2.05 (95% CI (1.69, 2.49), *P* < 0.001), this analysis provides an evidence that an increased TLR4 is a predictor of poor prognosis in patients with various cancers. In the subgroup, the adverse prognostic effect of high TLR4 on prognosis was significant in ethnicity, analysis type, cut-off value and HR obtained method. In the subgroup of tumor type, the association between high TLR4 expression and poor OS was significant, except for NSCLC. Thus, the effect of elevated TLR4 on prognosis in patients with lung cancers needs to be further confirmed. The elevated expression of TLR4 was demonstrated to be associated with the increased resistance to chemical treatments [46, 52]. Accumulating evidence suggested that high expression of TLR4 was associated with the metastasis of tumor and elevated TLR4 expression promotes tumor progression by contributing to metastasis. Numerous studies reported that the expression of TLR4 was associated with the metastasis of lymph nodes [18, 31, 32]. The evidence mentioned above may account for the association between the elevated TLR4 expression and poor prognosis of cancer patients. Moreover, our meta-analysis also demonstrated that the increased expression of TLR4 yielded a poor DFS (pooled HR = 1.79, 95% CI (1.11, 2.88), *P* < 0.05).

To the best of our knowledge, this is the first meta-analysis that systematically elucidates the prognostic value of TLR4 in various tumors. The evidence in our analysis demonstrated that increased expression of TLR4 predicted poor OS and DFS in patients with cancers. However, there are some limitations in our meta-analysis. First, the eligible studies included were only 15, and the sample size was relatively small, which leads to relatively insufficiency of data in the subgroup analyses. Second, since the lack of a unified cut-off value, various cut-off values of TLR4 expression were used in the enrolled studies. The inappropriate cut-off value may influence the capability of TLR4 to predict prognosis in patients with cancer. Third, most of the patients in the enrolled studies were Asian, and the applicability of the conclusion to Western patients should be questioned. The role of elevated TLR4 expression should be further investigated in western populations in future. Fourth, only the studies that were written in English were included, which may influence the robustness of the results. Fifth, unpublished literatures were not obtained and reviewed, which would likely include increased proportions of null results. Furthermore, although no significant difference was detected according to the results of sensitivity analysis and publication bias assay, publication bias cannot be totally ruled out because negative studies were not so acceptable as positive results. Finally, several HRs were obtained from the survival curves, and these data were less reliable than direct data from the original literature, which may inevitably bring about small deviations.

The TLR4 antagonist was reported to facilitate tumor reduction via enhancing apoptosis in colon cancer [53]. Yang et al. [54] reported that TLR4 antagonist suppress the invasiveness and migration of the human breast cancer cells. These findings demonstrated that TLR4 antagonists may have wide application prospect in defending against cancers or improving the prognosis.

In conclusion, the meta-analysis showed that increased expression of TLR4 is closely related to poor OS and DFS of patients with various tumors. The results indicate that TLR4, as a novel prognostic biomarker in solid tumors, could potentially help to improve treatment decision-making of solid tumors in clinical practice. Owing to the limitations of analysis, this conclusion should be regarded cautiously. Further researches with larger sample size are needed to confirm the prognostic effect of TLR4 on prognosis of patients and to explore more effective therapy strategies.

## MATERIALS AND METHODS

### Search strategy

Published articles that illustrated the role of TLR4 in cancer patients were searched through PubMed, Embase, and the Cochrane Library (last update by April 18, 2017). The key terms used in the process were “Toll Like Receptor 4 OR Toll-4 Receptor OR Toll 4 Receptor OR TLR4 OR TLR-4” (all fields) AND “cancer OR carcinoma OR tumor OR tumour OR neoplasm” (all fields) AND “prognosis OR prognostic OR survival OR outcome” (all fields). No advanced limitations were appended when searching the databases. The records of identified articles were also screened to further identify potential studies. Two reviewers carefully screened the literatures retrieved in the database.

### Inclusion and exclusion criteria

Studies that were qualified for inclusion in this meta-analysis according to the following criteria: (1) all patients enrolled were histopathologically confirmed the diagnosis of malignant disease; (2) investigation of the associations between TLR4 and survival outcome, including OS and DFS (3) Literatures provided prognostic HR or provided sufficient information that can calculate HR value.

Exclusion criteria: (1) articles that were not written in English; (2) case reports, meeting records, review papers, commentaries, clinical guidelines, or letters; (3) studies that didn’t provide important datum, such as HR or 95% CI; (4) studies on cancer cell lines and experimental animal researches; (5) studies of hematological malignancies were excluded. (6) no duplicate data. The same sample in multiple reports was enrolled only once.

### Data extraction and quality assessment

Two researchers collected the necessary information from all included articles independently, including first author’s family name, publication year, ethnic, cancer type, case number, tumor stage, the cut-off value, detected method, follow-up period, analysis type, and HR as well as corresponding 95% CI. If the statistical data were shown in the report, we extracted them directly. However, if an article did not provide HR and 95% CI, they were calculated using the data provided in the article. If only Kaplan-Meier curves of TLR4 were available, we were able to reconstruct the HRs and its 95% CIs from the data extracted from the curves according to the described method [55]. If both univariate and multivariate analysis for survival outcome were provided, only the multivariate was extracted since it has been more precise owe to accounting for confounding factors.

Two researchers assessed the quality of each study independently according to the NOS. The scores for quality assessment ranged from 0 (lowest) to 9 (highest), and studies with scores of 6 point or more were rated as high quality.

### Statistical analysis

Low and high expression of TLR4 was identified in accordance with the cut-off values provided in the articles. HRs and its 95% CIs were pooled to evaluate the value of increased level of TLR4 in prognosis of patients with solid cancers. If an HR > 1, it indicated a worse prognosis in patients with high expression of TLR4, and if HR < 1, it indicated a better prognosis. Statistical heterogeneity was evaluated by visual inspection of forest plots, by conducting the Chi-square test (assessing the *P* value), and by calculating the Higgins I-squared statistic [56]. The *P* < 0.05 and/or I^2^ > 50%, suggesting the presence of significant heterogeneity and a random-effects model (the DerSimonian-Laird method) should be conducted to calculate the pooled HRs. On the contrary, the fixed-effects model (the Mante-Haenszel method) should be used. Subgroup analyses were further conducted to test the source of heterogeneity. To investigate the potential source of heterogeneity among included studies, meta-regression was conducted using variables as ethnicity, cancer type, HR obtained method, analysis method and cut-off value. To validate the robustness of outcomes in this meta-analysis, sensitivity analysis was performed by sequential omission of each individual study. Publication bias was tested by assessing the asymmetry of a visual funnel plot. Also, we conducted Begg’s funnel plot and Egger’s linear regression test to evaluate publication bias. All statistical analyses were performed with STATA software version 12.0 (STATA Corporation, College Station, TX, USA) with significance defined as a *P* < 0.05 except where otherwise specified.
